# Topics of the Month

**Published:** 1893-04

**Authors:** 


### TOPICS OF THE MONTH.

One of the burning topics of the month in political and medical circles has been whether Dr. Bergtold would be appointed bacteriologist to the Health Department. At the last meeting of the Section of Medicine of the Buffalo Academy of Medicine, March 14, 1893, the following resolution was unanimously adopted:

> *Resolved,* That the Section on Medicine of the Buffalo Academy of Medicine hereby heartily approves the action of the Health Commissioner in his efforts to have a bacteriologist appointed by the city, considering as we do that in the present state of the medical science an expert is practically a necessity in properly guarding the public health.

The tenement-house question was also taken up, and a discussion arose on the advisability of adopting a resolution supporting the proposed ordinances respecting the matter. It was considered, however, that a better plan would be to appoint a committee to represent the Society at the meeting which was to have been held the following day, but which was postponed.

Drs. Lothrop, Pryor, and Rochester were placed on the committee.

---

At the commencement exercises of the Medical and Dental Department of the University of Tennessee, held in Nashville, Thursday evening, February 23d, the largest class was graduated that ever went forth from this institution. It was not only a large, but it was an especially bright, class, numbering among its members some of the most prominent young men of the South. Especially do we note that the first honors, including the Paul F. Eve faculty medal, was won by W. D. Haggard, Jr., son of Professor Haggard, in the University. It must be particularly gratifying both to father and son, when it is considered that young Dr. Haggard was the youngest member of the class, and has but just attained his majority. We feel assured that the honors have been worthily bestowed, and that in the future, should his life be spared, the profession of medicine will be adorned by the achievements of Dr. William D. Haggard, Jr.

---

THE Buffalo University Medical College dedicated its new building on Monday evening, March 6, 1893, in the presence of a large assemblage especially invited for the occasion. An historical address was made by Dr. Charles Cary, Professor of Materia Medica and Therapeutics, and the Vice-Chancellor, the Hon. James O. Putnam, delivered a speech preliminary to the delivery of Dr. Cary's address. After the exercises were concluded, the entire building was thrown open to the inspection of the guests, which is everywhere conceded to be one of the best medical college buildings in the country. Modern architecture has been invoked with reference to convenience as well as comeliness, and all the requisites of heating, lighting, ventilating, and plumbing, have been especially secured. In another department of the JOURNAL we publish Mr. Putnam's and Dr. Cary's addresses, which will be read with great interest by physicians and others interested in the prosperity of the school.

---

THE Riverside Hospital for Women, 2393 Niagara street, has a house staff composed of Dr. Lillian C. Randall and Dr. Mary S. Green, and is designed for the medical and surgical treatment of women. Physicians can find a pleasant home for patients upon whom they wish to operate. The consulting staff is Dr. Crockett, Dr. Putnam, Dr. Snow, Dr. Thompson, and Dr. Townsend. Terms will be furnished by Dr. Randall or Dr. Green.

---

THE January number of *The Bacteriological World and Modern Medicine* has donned a new dress, and made itself in other respects more attractive than ever. Drs. Kellogg and Paquin know evidently how to cater to the physician's wants, and have succeeded in making theirs, truly, a journal of "modern medicine."

---

THE New York *Polyclinic*, edited by the faculty of the New York Polyclinic School and Hospital, has made its appearance. It is a handsome journal, well edited, and, like its contemporary, the *Post-Graduate*, is filled with interesting and instructive matter. We see no reason why it should not receive the same hearty support as does the *Post-Graduate*.

